# Near-Infrared Spectroscopy Coupled with Chemometrics for Rapid Determination of pH, Moisture and Lycopene Content in Lycopene Liquid Beverages

**DOI:** 10.3390/foods15152676

**Published:** 2026-07-29

**Authors:** Xijie Zhao, Bingqian Hou, Ziyi Huang, Ni Qiu, Fengling Li, Jingjing Xia

**Affiliations:** School of Pharmaceutical Sciences, Institute of Materia Medica, College of Life Science and Technology, Xinjiang University, Urumqi 830017, China

**Keywords:** lycopene liquid beverage, near-infrared spectroscopy, chemometrics, variable selection, SHAP analysis

## Abstract

Lycopene liquid beverages are functional products with strong antioxidant activity, but their quality control is hindered by the lack of rapid and simultaneous detection methods for multiple indicators. This study investigated a near-infrared spectroscopy (NIRS)—based approach for the simultaneous quantification of pH, moisture, and lycopene content in lycopene liquid beverages. A combined preprocessing strategy integrating Savitzky-Golay smoothing and standard normal variate (SG-SNV) was optimized via grid search to reduce spectral noise and scattering effects. Model interpretability was further enhanced by SHapley Additive exPlanations (SHAP) analysis. Furthermore, four variable selection algorithms (MWPLS, UVE-SPA, ICO, and CARS) were compared to improve model performance and interpretability. The results showed that SG-SNV preprocessing notably improved the prediction performance for pH (R^2^_CV_ = 0.9398, R^2^_PRE_ = 0.9624) and lycopene (R^2^_CV_ = 0.9860, R^2^_PRE_ = 0.9576), while moisture prediction remained at a comparable level (R^2^cv = 0.9617, R^2^_PRE_ = 0.9727). SHAP analysis identified spectral regions that may be associated with each component, providing chemically plausible explanations. For variable selection, the optimal algorithm differed across the three quality indicators. For moisture, CARS achieved the highest prediction performance (R^2^_PRE_ = 0.9946), while ICO provided comparable accuracy with slightly better model stability. For pH, UVE–SPA produced the strongest generalization ability (R^2^_PRE_ = 0.9705) with only 5 selected variables. For lycopene, none of the variable selection methods improved upon the preprocessing-only model (R^2^_PRE_ = 0.9576); MWPLS retained a parsimonious 21-variable model but with reduced predictive accuracy (R^2^_PRE_ = 0.8888). This study explores a preliminary rapid and non-destructive NIR analytical approach for the simultaneous detection of multiple quality indicators in lycopene liquid beverages, suggesting the feasibility of chemometric modeling for this application. The results provide a preliminary foundation for future development of rapid multi-indicator analytical methods. However, this work was conducted entirely under controlled laboratory conditions using a small, gradient-designed sample set with no production-line or online validation; it should therefore be regarded as a proof-of-concept feasibility study. Substantial further work—including validation with larger, naturally variable sample cohorts—would be needed before the approach could be considered for practical deployment.

## 1. Introduction

Lycopene, a natural highly conjugated polyene carotenoid, exhibits strong free radical scavenging and antioxidant activities. It has been shown to effectively delay vascular endothelial aging, improve lipid metabolism, and modulate immune responses [1]. As humans cannot synthesize lycopene endogenously, preparing it as a liquid beverage is a key approach to enhance dietary intake and promote its functional effects [2,3]. However, quality control of lycopene liquid beverages remains predominantly reliant on traditional analytical methods. For instance, thin-layer chromatography involves cumbersome procedures, and its quantitative accuracy fails to meet the requirements for quality control [4,5]. High-performance liquid chromatography, regarded as the classic gold standard for lycopene detection, offers excellent separation efficiency. However, it necessitates complex pretreatment steps, including extraction with n-hexane-acetone mixtures and purification via solid-phase extraction. These procedures not only incur high detection costs but also face limitations in method applicability due to the altered spectral characteristics of lycopene liquid beverages [6,7,8,9].

Near-infrared spectroscopy (NIRS) is a green, efficient, and non-destructive analytical technique that indirectly retrieves the chemical composition and physical properties of a sample by detecting the overtone and combination absorption signals of hydrogen-containing groups (e.g., C-H, O-H, N-H) in organic molecules [10,11]. Leveraging its core advantages, such as rapid detection, non-destructive analysis, minimal sample pretreatment, simultaneous quantification of multiple components, and potential compatibility with online detection workflows, this technology has been widely adopted and applied in the field of food and pharmaceutical quality analysis [12,13,14]. However, successful online deployment requires instrument adaptation, environmental control, and extensive field validation beyond laboratory proof-of-concept studies; the present study falls within this early-stage exploration, and no online or production-line validation has been conducted.

Previous studies have demonstrated that NIRS can effectively classify liquid foods, including fruit wines and liquid milk. The technique achieves this by accurately distinguishing spectral differences among samples [15,16]. Recent work has extended NIRS beverage analysis in several key ways: from binary classification to simultaneous multi-component quantification, from traditional linear regression to machine learning algorithms such as BPNN and ANN, and from simple beverages to complex functional drinks. For example, Rácz et al. quantified caffeine and sugar in commercial energy drinks using FT-NIR with PLSR (R^2^ > 0.90) [17], while Bai et al. simultaneously predicted four quality parameters in instant tea using BPSO-optimized models (Rp up to 0.9757) [18]. Wang et al. systematically compared six variable selection methods and six machine learning algorithms for polysaccharide determination in shiitake mushroom beverage, achieving Rp^2^ ≥ 0.98 with the SG-BPNN model [19]. Elamshity and Alhamdan demonstrated that ANN outperformed PLSR (R^2^ 0.946–0.989) in predicting properties of date-syrup-flavored milk drinks using VIS-NIR [20]. Shen et al. further showed that NIRS with OPLS-DA could simultaneously discriminate coconut water by storage time, cultivar, and maturity with >95% accuracy [21]. These advances collectively illustrate the progression toward multi-component, multi-factor, and machine-learning-driven NIRS analysis of increasingly diverse beverage matrices.

However, due to the severe spectral overlap and discontinuity inherent in the near-infrared region, the component-related information embedded in NIR spectra is difficult to extract directly and to interpret with reasonable spectral assignment. Consequently, NIR spectral analysis is often combined with chemometric methods to optimize model performance, employing strategies such as combined preprocessing and variable selection. Existing studies have found that a single preprocessing method can rarely reduce these multiple interferences reliably. Mishra et al. point out that a complementary combination of multiple preprocessing methods can utilize useful information more fully than a single correction method and can reduce the reliance on manual, repetitive screening of preprocessing workflows [22]. For instance, Chen et al. combined nonlinear background correction, Lorentzian peak fitting, and multi-feature machine learning regression. This approach effectively reduced the quantification error, achieving a root mean square error (RMSE) of 5.50 mg/dL and a coefficient of determination (R^2^) of 0.999 [23]. Xiao et al. combined first derivative with SNV and incorporated deep transfer learning. This approach enabled the transfer of leaf chlorophyll content estimation models across different cotton varieties [24]. Qi et al. combined continuous wavelet transform (CWT), feature selection, and machine learning for wheat gluten strength type identification. They identified that a ReliefF-CWT combined with support vector machine (SVM) achieved high classification accuracy. Their results indicated a synergistic relationship among preprocessing, feature selection, and classification models [25]. Variable selection could remove uninformative and interfering variables, retain characteristic wavelengths related to the target component, and thereby simplify the model, improve prediction accuracy, and enhance interpretability [26]. For example, Yu et al. compared three variable selection methods, including Competitive Adaptive Reweighted Sampling (CARS), uninformative variable elimination (UVE), and Random Frog (RT), and found that the CARS-CWT-PLS model delivered the best performance, achieving a correlation coefficient of 0.9700 and an RPD of 5.229 [27]. Xia et al. used NIRS together with three variable selection algorithms, SPA, genetic algorithm–partial least squares (GA–PLS), and CARS, to select optimal characteristic wavelengths. Results indicated that the combination of CARS with artificial neural network model gave the best performance [28].

Against the above research background, this study selected different batches of lycopene liquid beverages and determined their multiple component indicators, including lycopene content, pH, and moisture. NIRS was employed to construct predictive models for these components. The novelty of this work is fourfold. First, to the best of our knowledge, this is the first study to apply NIRS for the characterization of lycopene-based liquid beverages, extending the technique to a functionally important but analytically less-studied matrix. Second, we simultaneously predict three quality indicators, lycopene content, pH, and moisture, within a unified spectral modeling framework, rather than targeting a single component as in most prior beverage studies. Third, we introduce SHAP analysis to interpret the chemometric models, providing data-driven insight into which spectral regions drive the predictions for each component, which has not been widely explored in NIRS-based beverage analysis. Fourth, we systematically compare multiple variable selection strategies to identify the characteristic spectral regions for each quality indicator, thereby enhancing both model parsimony and interpretability. The objective of this study is to optimize raw spectral data, extract critical features, and achieve preliminary simultaneous prediction of multiple components under laboratory conditions, thus exploring the feasibility of a NIR-based detection approach for the multi-indicator analysis of lycopene-based liquid beverages.

## 2. Materials and Methods

### 2.1. Sample Preparation

Construction of continuous concentration gradients: To achieve simultaneous and accurate prediction of key indicators (lycopene, pH, and moisture content) in the samples, we used the tomato-based liquid drink stock solution produced by Xinjiang Tianshan Shenghe Biotechnology Co., Ltd. (Turpan, China) as the base material. Eight independent production batches of this stock solution were selected as parent materials. A series of concentration gradients was prepared from each batch. The final sample set consisted of 8 undiluted stock samples, 8 half-diluted (1/2) samples, 8 quarter-diluted (1/4) samples, 7 two-fold concentrated samples, and 5 four-fold concentrated samples. This yielded a total of 36 samples for model development.

pH and moisture content: Sample pH was measured using a PHSJ-3F high-precision pH meter (manufactured by Shanghai Yidian Scientific Instrument Co., Ltd., Shanghai, China), which was calibrated with standard buffer solutions. The meter was used for direct injection measurement to ensure stable readings. The moisture content in the continuous phase was determined using an SH10A halogen rapid moisture analyzer (manufactured by Shanghai Jinghai Instrument Co., Ltd., Shanghai, China). After instrument baseline calibration, a dedicated test mode was activated. The absolute percentage of moisture was then output based on the principle of efficient thermogravimetric loss.

Lycopene content: Samples were collected from each concentration gradient and from multiple batches of conventional products. Lycopene quantification was performed using a spectrophotometric method, as lycopene possesses a conjugated polyene chromophore that exhibits well-defined absorption maxima in the visible region, with characteristic peaks near 472 nm and 504 nm. Notably, carotenes display their maximum absorption at 420 nm and 444 nm, thereby causing no spectral interference under the measurement conditions [29]. Compared with chromatographic techniques such as HPLC, this method offers a simpler and more rapid analytical workflow, and has been extensively documented in the literature for the analysis of lycopene in tomato-based matrices [29,30,31]. Thirty-six lycopene liquid beverage samples were vortexed (or shaken) to ensure homogeneity. Aliquots of 100 μL from each sample were pipetted into separate wells of a 96-well plate. The plate was subsequently loaded into a microplate reader, and the absorbance was recorded at 472 nm against a blank control. The obtained measurement results were directly applied to a pre-validated lycopene reference standard curve (regression equation: Y = 126.42X + 0.0571, coefficient of determination R^2^ > 0.99). The true lycopene concentration in each corresponding sample was then calculated. During the measurement process, three technical replicates were set for each parallel sample to reduce random operational errors.

Lycopene reference standard curve: Using lycopene reference standard (Beijing Solarbio Science & Technology Co., Ltd., Beijing, China; purity ≥ 98%), dichloromethane containing 0.05% BHT was used as the solvent to prepare a standard stock solution at a concentration of 0.2 mg/mL under light-protected conditions. This stock solution was further diluted to obtain a series of standard working solutions at concentrations of 0, 0.004, 0.008, 0.012, 0.016, and 0.020 mg/mL. Using the blank solvent as the reference, the absorbance of each concentration point was measured at a wavelength of 472 nm, with three replicates taken for each and the mean values recorded as 0.056452, 0.573067, 1.051033, 1.570967, 2.100400, and 2.575850, respectively. Linear regression was performed with concentration as the abscissa (X, mg/mL) and absorbance as the ordinate (Y), yielding the standard curve equation Y = 126.42X + 0.0571, with a correlation coefficient R^2^ = 0.9998.

The total sample size for this study was set at 36. The aim was to assess whether NIR spectroscopy combined with chemometric models can simultaneously detect multiple quality indicators in lycopene liquid beverages. We used a gradient design that covered five concentration levels and eight production batches. The analytical range spanned from fourfold dilution to fourfold concentration, to achieve broad coverage. The design emphasised internal chemical variability rather than natural sample diversity. Although 36 samples are insufficient for building a robust, generalisable industrial model, this study may serve as a methodological feasibility exploration.

### 2.2. Near-Infrared Spectra

A TIGER-T Fourier transform near-infrared spectrometer was used throughout the spectral acquisition process. It was equipped with an infrared halogen light source, an InGaAs solid-state detector, and a high-efficiency quartz beam splitter. The test window covered a wide wavenumber range from 12,000 to 4000 cm^−1^. The spectral resolution of the instrument was 8 cm^−1^, and the average number of scans per sample was 32. Spectra were acquired using a cylindrical sample cell with an internal diameter of 8 mm. All measurements were performed at room temperature without active temperature control.

Prior to sampling, each gradient lycopene liquid beverage sample was manually shaken to ensure homogenization and minimize the influence of suspended particles, bubbles, and settling. The homogenized sample was then injected into the sample cell, and the liquid level was carefully ensured to completely cover the detection spot, thereby minimizing stray light interference. Each prepared sample was repeatedly loaded and independently scanned three times, with the sample cell cleaned with ethanol between successive samples. A background spectrum was acquired once every hour. The spectra of all samples were acquired in randomized order. Finally, the mathematical average curve of the three repeated spectra was extracted as the unique raw spectral representation for that sample, thus ensuring spectral stability.

### 2.3. Spectral Preprocessing

Instrumental noise is unavoidable during NIR spectral acquisition. Savitzky-Golay (S-G) smoothing reduces random noise through polynomial fitting within a local window. It smooths the spectral curve while largely preserving the original peak shapes and positions [32]. Since the particles in lycopene liquid beverages are micrometer-sized or larger, NIR spectral acquisition is inevitably affected by light scattering. Standard normal variate (SNV) centers and scales each individual spectrum. This process reduces intensity shifts caused by variations in optical path length, changes in dispersion state, and particle scattering [33]. Therefore, in this study, based on the NIR spectral characteristics of lycopene liquid beverages, a combined preprocessing strategy of S-G smoothing and SNV was adopted. The two parameters of S-G smoothing, polynomial order and window size, were optimized using a grid search approach based solely on the training set to avoid data leakage into the test set. In this study, the polynomial order was set to 1, 2, and 3. For moisture content, pH, and lycopene content, we adopted the same set of window sizes: 5, 7, and 9.

### 2.4. Variable Selection Methods

To improve model performance, we adopted variable selection algorithms grounded in different principles to optimize the model results. The algorithms include the Uninformative Variable Elimination–Successive Projections Algorithm (UVE–SPA) for eliminating irrelevant information, CARS for optimizing point combinations, Interval Combination Optimization (ICO) for combining intervals, and Moving Window Partial Least Squares (MWPLS) for selecting individual intervals. In brief, ICO divided the full spectrum into several equally wide sub-intervals. A PLSR model is built for each sub-interval. The optimal sub-intervals are selected based on their predictive performance. The wavelength variables from these optimal sub-intervals are then combined and used as the selected feature variables [34]. UVE–SPA is a hybrid variable selection method. First, UVE removes noise variables that do not contribute to the model based on the statistical stability of their regression coefficients. Then, the successive projections algorithm (SPA) is applied to the remaining variables to further reduce collinearity. This process yields a combination of feature variables that contains minimal redundant information [35]. CARS performs iterative adaptive reweighted sampling and uses the absolute values of regression coefficients from PLSR. Irrelevant variables with low weights are successively eliminated, and characteristic wavelengths with the highest regression coefficient weights are ultimately retained. During the selection process, the method preserves a small number of key variables that are highly correlated with the target analyte [36]. MWPLS is a variable selection method that operates with a fixed-width window. The window is moved point by point across the entire spectral wavelength range. At each window position, a PLSR model is built, and the prediction errors of the models corresponding to different windows are compared. The wavelengths within the window that yields the lowest prediction error are finally selected as the feature variables [37].

### 2.5. SHAP Analysis

SHAP analysis originates from Shapley values in cooperative game theory, and was later systematized and popularized for interpreting machine learning (ML) models by Lundberg et al. [38]. At its core, SHAP treats model features as “players” in a cooperative game, fairly distributing each feature’s marginal contribution to every individual prediction. SHAP values inherit four key properties from Shapley values: efficiency, symmetry, additivity, and the null player property. Among these, efficiency is the most critical—the sum of all feature SHAP values for a single sample equals the difference between that sample’s prediction and the overall mean prediction, which allows SHAP values to be expressed directly in the units of the prediction. For interpretation, a hierarchical analysis workflow is recommended [39]. First, SHAP values should be computed on the test set only after the model demonstrates adequate performance, and their consistency should be verified across k-fold cross-validation. Next, the bar plot provides a global ranking of feature importance by mean absolute SHAP value. The beeswarm plot then reveals the directionality, non-linearity, and outliers of each feature’s impact. The scatter plot further enables in-depth inspection of individual feature–SHAP relationships, where the vertical spread of SHAP values at a given feature value signals interaction effects with other features. Finally, the waterfall plot supports local, case-level interpretation for individual samples [39]. For classification tasks, SHAP values should be examined on both the probability scale and the log-odds scale: the former facilitates quantitative communication, while the latter is better suited for inferring linear relationships. Two fundamental limitations should be noted. First, SHAP values may be distorted under high feature correlation, in which case conditional SHAP or prior feature de-redundancy should be considered. Second, SHAP values quantify how features contribute to the model’s predictions, not their causal effects in the real world. Building on these foundations, Bernal et al. further proposed combining SHAP value analysis with raw feature value range analysis to construct “RAW” and “SHAP” joint flagging rules [40]. When a sample’s feature values (or SHAP values) frequently fall within the typical ranges of the opposite class, the prediction carries a high risk of misclassification. This approach forms a post-prediction screening framework that can effectively reduce false positives and false negatives in applications such as virtual screening.

SHAP analysis was performed using PLS regression as the modeling approach. The model, an sklearn.cross_decomposition. PLSRegression instance (optimal_plsr), was trained with the optimal number of latent variables determined via 10-fold cross-validation. SHAP values were computed using the Permutation Explainer method implemented in the Python 3.14 shap library. This method does not require a background dataset; instead, it evaluates feature importance by randomly permuting the values of each feature and measuring the consequent degradation in model predictive performance, rendering it well-suited for global importance assessment of spectral variables. An explainer was constructed using shap.Explainer with max_evals set to 1503, corresponding to the default of 10 permutation evaluations per wavelength variable across the 1503 variables. The SHAP explanation was generated based on all training set samples. The SHAP value matrix was subsequently obtained by calling explainer.shap_values, while the shap.Explanation object was concurrently generated via the explainer. The above analysis was conducted on the pre-processed full-spectrum PLS model (SG smoothing combined with SNV transformation), incorporating all 1503 wavelength variables as input features to evaluate the contribution of each wavelength to model predictions. Given the severe multicollinearity among adjacent spectral variables, the permutation-based strategy of the Permutation Explainer can, to some extent, mitigate the influence of correlated variables on importance allocation. In parallel, the PLS model inherently compresses correlated variables through latent variable projection. Finally, feature importance was visualized as a bar plot using the shap.summary_plot function.

### 2.6. Modeling Methods and Model Evaluation

In regression models, the R^2^ is commonly used to evaluate the goodness of fit between observed and predicted values. Its value ranges from 0 to 1. An R^2^ close to 1 indicates that the model explains most of the variation in the observed data. Conversely, an R^2^ close to 0 suggests a poor model fit [41].

$$R^{2}=1-\frac{\Sigma_{\mathrm{i}=1}^{n}{\left(y_{i}-{\hat{y}}_{i}\right)}^{2}}{\Sigma_{\mathrm{i}=1}^{n}{\left(y_{i}-\bar{y_{i}}\right)}^{2}}$$

The RMSE is another important metric for evaluating model prediction accuracy. It reflects the average deviation between predicted and observed values [42]. RMSE is more sensitive to large errors. A smaller RMSE value indicates higher prediction accuracy.

$$\mathrm{RMSE}=\sqrt{\frac{1}{n}\Sigma_{\mathrm{i}=1}^{n}{\left(y_{i}-{\hat{y}}_{i}\right)}^{2}}$$

The ratio of performance to deviation (RPD) and the range error ratio (RER) are used to assess the relationship between model prediction error and the inherent variability of the data. RPD evaluates model performance as the ratio of the standard deviation to RMSE, while RER uses the ratio of the data range to RMSE. Generally, higher RPD or RER values indicate that the model error is small relative to data variation, suggesting superior predictive ability. It is generally accepted that a model has good predictive ability and can be considered adequate for screening-level applications when RPD ≥ 2.5 or RER ≥ 10 [43]. However, with only 9 samples in the independent test set, the confidence intervals of these ratio-based metrics are necessarily wide, and the corresponding threshold judgments should be interpreted with appropriate caution.

$$\mathrm{RPD}=\frac{\mathrm{SD}}{\mathrm{RMSE}}$$

$$\mathrm{RER}=\frac{y_{\max}-y_{\min}}{\mathrm{RMSE}}$$

In the above formulas, n is the number of samples, $y_{i}$ denotes the observed value, $\bar{y_{i}}$ represents the mean of the observed values, ${\hat{y}}_{i}$ denotes the predicted value, SD is the standard deviation of the observed values, and $y_{\max}\;\mathrm{and}\;y_{\min}$ represent the maximum and minimum observed values, respectively.

Each batch was scanned in triplicate, and the average spectrum was taken as its representative spectrum. A systematic sampling approach was used. One batch from every four consecutive batches was assigned to the test set, while the other three were assigned to the training set. This yielded 27 training samples and 9 test samples. The optimal PLSR parameters were determined via 10-fold cross-validation. All data processing and modeling were carried out with MATLAB R2024a.

## 3. Results and Analysis

### 3.1. Chemical Values

**Figure 1:**
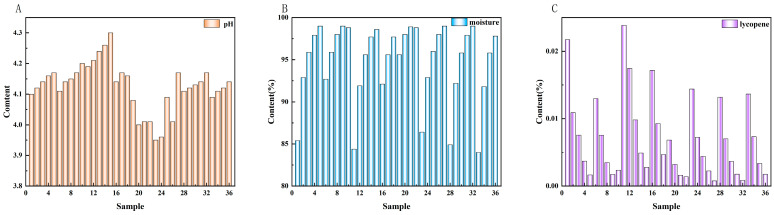
Histograms showing the distribution of pH, moisture content, and lycopene content in 36 batches of lycopene liquid beverage samples: (**A**) pH; (**B**) Moisture content; (**C**) Lycopene content.

In this study, 36 Lycopene liquid beverages were used. NIRS was employed to determine the contents of pH, moisture, and lycopene (Table 1). The pH of the samples ranged from 3.95 to 4.26 with a mean value of 4.12 and a standard deviation of 0.07, indicating highly consistent pH across the samples (Figure 1A). The moisture content ranged from 84% to 99.0%, with a mean of 94.78% and a standard deviation of 4.6%. The overall variability was small (Figure 1B). The lycopene content ranged from 0.0753% to 2.3864%, with a mean of 0.7176% and a standard deviation of 0.6104%. These results indicated that lycopene content varied substantially among the samples, showing high dispersion and a distinct concentration gradient (Figure 1C). This variation provides a sufficient concentration range for exploratory NIR quantitative modeling under the current experimental design. The data exhibited good stability and can provide a basis for subsequent NIR modeling.

### 3.2. Analysis of Near-Infrared Spectra

**Figure 2:**
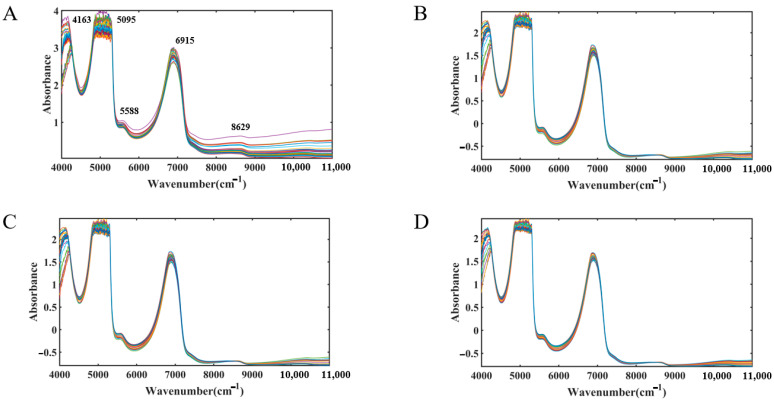
Comparison of spectra before and after pretreatment. (**A**) Raw spectra. (**B**) Spectra after S-G smoothing. (**C**) Spectra after SNV. (**D**) Spectra after S-G smoothing and SNV.

Figure 2 shows the raw NIR spectra of lycopene liquid beverages and the positions of their characteristic absorption peaks. From Figure 2A, five distinct absorption peaks can be observed near 4163, 5095, 5588, 6915 and 8629 cm^−1^. The peak around 4163 cm^−1^ is partially related to the combination band of C-H and C=C, which may be associated with the conjugated double bond structure of lycopene molecules as well as C–H vibrations from other hydrocarbon components in the beverage matrix [44]. The peak around 5095 cm^−1^ may be attributed to the combination band of O-H stretching and bending vibrations, likely dominated by water molecules as the major absorbing species in this aqueous matrix [45]. The peak at 6915 cm^−1^ may primarily arise from the first overtone of the O-H stretching vibration of water molecules, though contributions from other hydroxyl-containing solutes cannot be excluded [45]. The peak at 5588 cm^−1^ may be attributed to the first overtone of C-H stretching vibrations [46]. The peak at 8629 cm^−1^ may be attributed to the second overtone of C-H stretching vibrations [46]. It should be noted that these assignments are based on general NIR literature; in a complex aqueous beverage matrix, signals from water and other organic constituents may overlap with or dominate the features described above. Although these characteristic peaks provide a basis for component identification, the spectra exhibit high similarity and significant peak overlap. They are also affected by noise and environmental factors. Therefore, further analysis using appropriate spectral preprocessing methods and chemometric techniques is necessary.

This study applied the S-G-SNV combined preprocessing method to the raw spectral data for pH, moisture content, and lycopene content, respectively. The preprocessed spectra are presented in Figure 2B–D. After S–G smoothing, the characteristic peak positions show no noticeable shift. High-frequency noise is substantially reduced, and the spectral curves become smoother. Following SNV pretreatment, scattering and baseline drift are reduced. The absorbance response range broadens, the contrast of the characteristic peaks is enhanced, and physical interferences are mitigated. As a result, the relative differences among characteristic peaks are more clearly revealed, making the spectral variations across samples more apparent for subsequent modeling.

As shown in Table 2, the PLSR models built from raw spectra exhibited substantial differences in prediction performance among the quality attributes. The moisture content model performed the best, with training and test coefficients of determination of 0.9898 and 0.9951, respectively, and RPD values both above 6, indicating high accuracy and stability. In contrast, the pH model showed weaker performance: its R^2^_CV_ and R^2^_PRE_ were 0.7592 and 0.9276, and the corresponding RPD values were only 2.0767 and 3.9424. These results suggest limited predictive power, restricting the model to rough quantitative analysis. The lycopene model exhibited intermediate performance. Although both the training and test R^2^ values exceeded 0.85, the RPD values ranged from 3 to 3.2, implying that prediction accuracy can still be improved. Overall, while the raw spectra can capture sample information, the models varied in stability and accuracy. This variability provides a necessary basis for subsequent spectral preprocessing and feature optimization.

**Figure 3:**
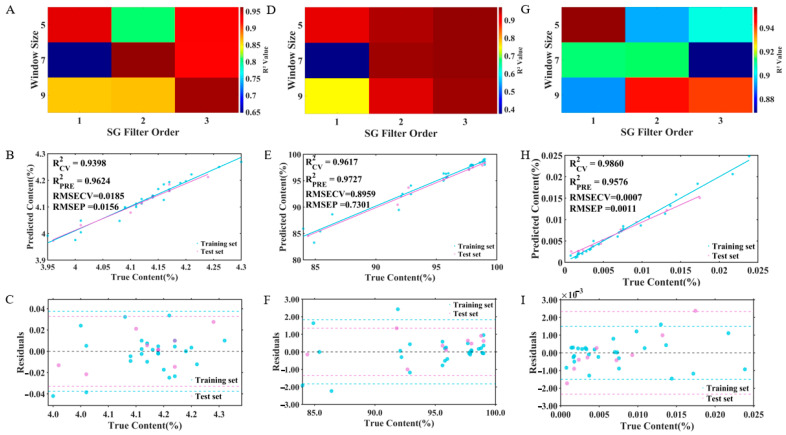
Regression plot and residual plot of the optimal model. (**A**–**C**) represent the heatmap optimization process, regression plot and residual plot for pH. (**D**–**F**) represent the heatmap optimization process, regression plot and residual plot for moisture content. (**G**–**I**) represent the heatmap optimization process, regression plot and residual plot for lycopene.

We conducted a heatmap analysis to exhaustively compare different Savitzky–Golay (S-G) filter parameters (window size and polynomial order), using the R^2^ as the optimization metric. In the heatmaps, colors approaching dark red indicate higher R^2^ values and better model performance. For the pH indicator, the highest R^2^ was obtained with a window size of 7 and a polynomial order of 2 (Figure 3A). Compared with the original model, the SG-SNV combined preprocessing notably improved the pH and lycopene models but not the moisture prediction, yet the moisture prediction model maintained a high level of performance (Table 3). For the pH model (Figure 3B), the R^2^ of the training set (R^2^_CV_) increased from 0.7592 to 0.9398, and that of the test set (R^2^_PRE_) increased from 0.9276 to 0.9624. Both RMSECV and RMSEP decreased. Figure 3C presents the residual distribution between measured and predicted pH values. The residual distribution of pH between the training and test sets is depicted. Most residuals lie within ±0.04. However, one residual from the test set falls outside this interval (Figure 3C). These results indicate that the preprocessing reduced noise and scattering effects. Moisture content exhibited generally high R^2^ across all parameter combinations; the combination of window size 7 and order 3 performed best and was therefore adopted as the optimal model (Figure 3D). The original results of the moisture model were already good. After preprocessing, the R^2^_CV_ and R^2^_PRE_ both decreased slightly (Figure 3E). The residual distribution for moisture content reveals that two samples from the training set fall outside the ±2.00 boundary (Figure 3F). Lycopene content yielded its maximum R^2^ at a window size of 5 and order 1 (Figure 3G). The optimal filter parameter combinations for the three indicators were all different, highlighting their distinct sensitivities to the degree of spectral smoothing and fitting complexity. In addition, the lycopene content model showed the most notable improvement. The R^2^_CV_ increased from 0.8863 to 0.9860, and the RPDc reached 8.6277. The R^2^_PRE_ of the test set also increased from 0.8890 to 0.9576 (Figure 3H). These results indicate that both the model fitting ability and the prediction ability were enhanced. For lycopene content, the residual plot (Figure 3I) shows the distribution of actual versus predicted values. Only one test set sample exceeds the ±2.00 × 10^−3^ limits. Therefore, the model generalization ability should be further evaluated using an independent test set. Overall, the SG-SNV combined preprocessing can improve the quality of spectral information, and its optimization effect was more pronounced for the pH and lycopene content models.

### 3.3. SHAP Feature Contribution Analysis

**Figure 4:**
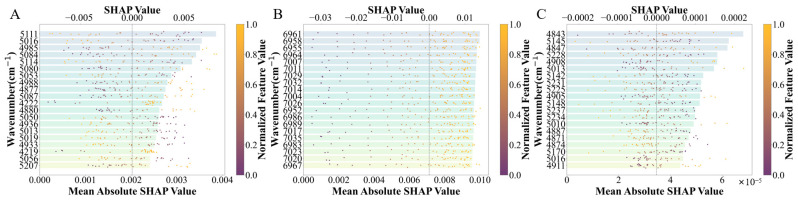
The SHAP bee swarm plots and scatter plots for each component. (**A**) pH; (**B**) moisture; (**C**) lycopene.

To further enhance the interpretability of the model, SHAP analysis was performed. Figure 4A shows the top 20 features contributing to the prediction of pH. The corresponding wavenumbers are 5111, 5016, 4985, 5084, 5114, 5080, 5053, 4988, 4877, 5087, 4222, 4880, 5050, 4936, 5013, 5019, 4933, 4219, 5056 and 5207 cm^−1^. Wherein, spectral bands such as 5114, 5050, 4936, 5013, and 4219 cm^−1^ showed a notable positive correlation with pH. In contrast, bands such as 5207, 5056 and 4933 cm^−1^ showed a negative correlation. Key absorption peaks around 5200 cm^−1^ (e.g., 5207 cm^−1^) may be tentatively assigned to the combination bands of O-H stretching and bending vibrations, consistent with water as the predominant absorbing species in this region [47,48]. However, in complex aqueous beverage matrices, overlapping signals from carboxylic acids and other hydroxyl-bearing constituents may also contribute to absorption in this spectral range, and definitive assignment to a single species is not warranted solely from SHAP feature importance. Figure 4B shows the top 20 features contributing to the prediction of moisture. The corresponding wavenumbers are 6961, 6958, 6955, 6964, 7007, 7011, 7029, 7032, 7014, 7004, 7026, 6952, 6986, 6989, 7017, 7035, 6983, 7023, 7020 and 6967 cm^−1^. Among these variables, the high-wavenumber bands centered at 6961, 6958, 6955, 6964 and 7007 cm^−1^ show dominant positive SHAP values, indicating that these spectral regions contribute positively to the moisture content prediction. These bands may be tentatively attributed to overtone and combination absorption features associated with O–H stretching vibrations, likely reflecting contributions from both bound and free water molecules as well as other hydroxyl-bearing matrix constituents. In contrast, the bands around 7020, 7023, 6983, 7035 and 7017 cm^−1^ present relatively lower SHAP contributions, implying a weaker or opposite influence on the model output, which may be related to the complex coupling effects between water-associated hydroxyl groups and the surrounding matrix [48,49,50]. Similarly, Figure 4C shows the top 20 features contributing to the prediction of lycopene. Observing the distribution of feature impacts, spectral bands such as 4843, 4847, and 4871 cm^−1^ showed a positive correlation with target concentration. In contrast, bands such as 5145 and 5228 cm^−1^ showed a negative correlation. Key absorption peaks in the range of 4000–5128 cm^−1^ (e.g., 4843, 4847, 4908, 5013 and 4905 cm^−1^) may be tentatively attributed to the combination bands of C-H stretching and bending vibrations in organic molecules [49]. These peaks are plausibly associated with the skeletal vibrational features of organic constituents in the beverage matrix, though the specific contribution from lycopene versus other hydrocarbon-containing species cannot be resolved from SHAP analysis alone. Absorption peaks in the range of 4800–4950 cm^−1^ (e.g., 4843, 4847 and 4871 cm^−1^) may be tentatively assigned to the combination bands related to O-H or specific C–H groups in the target and its micro-environment [49]. Notably, these bands fall in a spectral region that is less dominated by the strong water absorption near 5150 cm^−1^, which may reduce spectral interference from the aqueous background. The changes in their SHAP values suggest a correlation with the target concentration, although contributions from co-varying matrix components cannot be entirely ruled out.

### 3.4. Variable Selection

Full-spectrum models are easily affected by redundant spectral information, which compromises prediction stability. Therefore, this study applied four variable selection methods—MWPLS, UVE–SPA, ICO, and CARS—to identify characteristic wavelengths for moisture quantification from the near-infrared spectra of lycopene liquid beverages.

**Figure 5:**
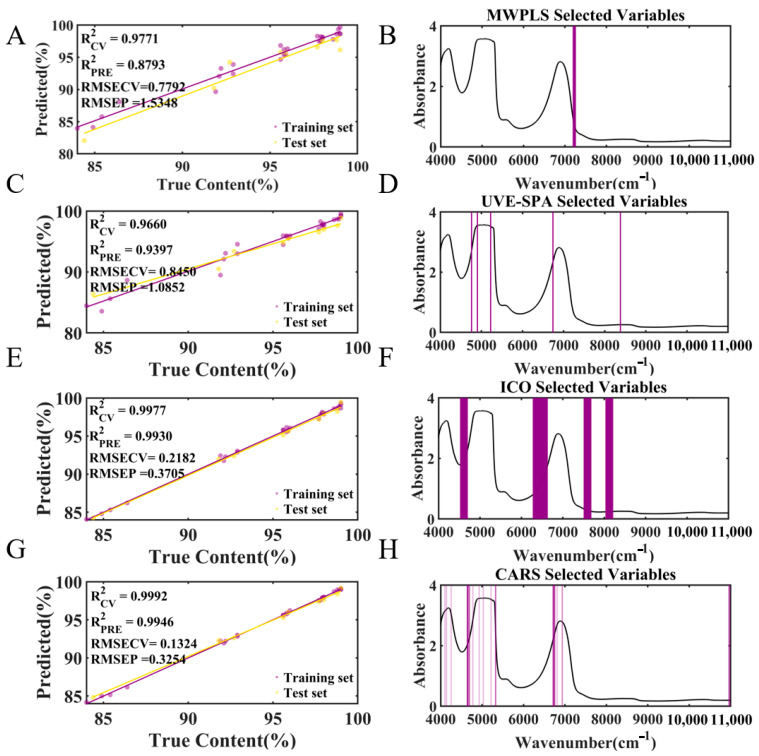
The regression fitting plots for the water model and the variable selection regions. (**A**,**B**) display the fitting plot and the corresponding variable selection region for MWPLS, (**C**,**D**) for UVE-SPA; (**E**,**F**) for ICO; and (**G**,**H**) for CARS.

As shown in Figure 5A,B, MWPLS identified 21 characteristic variables. The R^2^cv was 0.9771, indicating good internal fit; however, the R^2^_PRE_ was only 0.8793, revealing limited generalization ability and pronounced overfitting. Consequently, this model showed limited prediction stability within the current dataset. As shown in Figure 5C,D, UVE–SPA selected only 5 characteristic variables. The R^2^cv was 0.9660, and the R^2^_PRE_ reached 0.9397, indicating a marked improvement in both stability and prediction accuracy. The model showed relatively stable prediction performance on the current test set. As shown in Figure 5E,F, ICO identified 285 characteristic variables. The R^2^cv reached as high as 0.9977, and the R^2^_PRE_ was 0.9930, indicating excellent fitting precision combined with strong generalization ability. The overall performance of ICO was the best among the four methods under the current experimental conditions. As shown in Figure 5G,H, CARS identified 37 characteristic variables. Its R^2^cv reached 0.9992, indicating the best internal fit; however, the R^2^_PRE_ was 0.9946, showing a slight overfitting tendency and slightly weaker generalization stability than ICO. Nevertheless, CARS still showed high prediction accuracy on the current test set, although its robustness requires further confirmation.

In summary, UVE–SPA, ICO, and CARS can all improve the performance of the NIR quantitative models for moisture under the current experimental conditions. Among them, the CARS model exhibits the highest internal fitting accuracy, whereas the ICO model achieves the most favorable balance among fitting accuracy, predictive ability, and model complexity, with a lower risk of overfitting. Specifically, ICO identifies four characteristic water bands: 4500–4700 cm^−1^, 6250–6650 cm^−1^, 7500–7700 cm^−1^, and 8000–8250 cm^−1^. These bands may correspond to overtones and combination bands of O–H stretching and bending vibrations in water molecules. They may also be related to O–H stretching vibrations of hydroxyl groups in the sample matrix.

**Figure 6:**
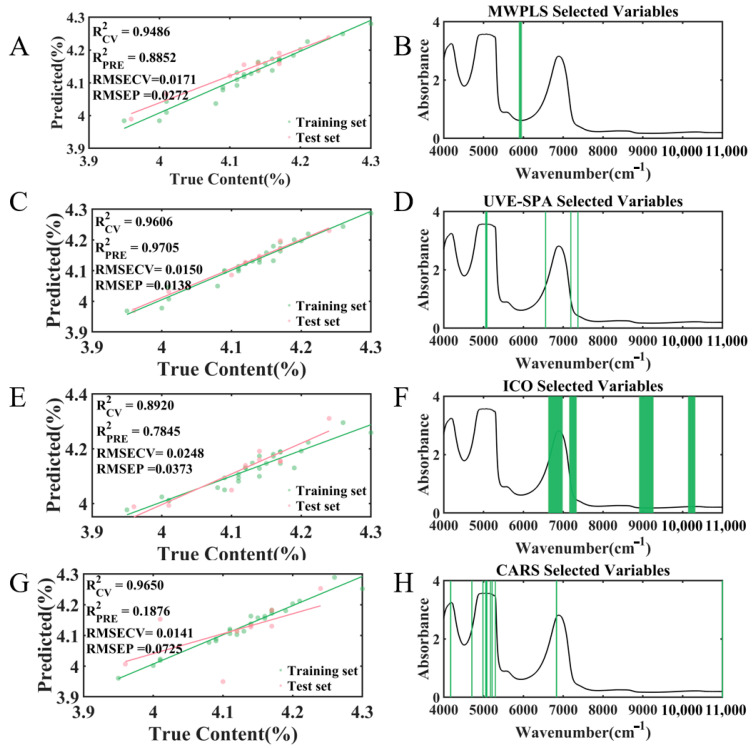
The regression fitting plots for the pH model and the variable selection regions. (**A**,**B**) display the fitting plot and the corresponding variable selection region for MWPLS, (**C**,**D**) for UVE-SPA; (**E**,**F**) for ICO; and (**G**,**H**) for CARS.

As shown in Figure 6A,B, MWPLS selected 21 characteristic variables. The R^2^cv was 0.9486, indicating acceptable internal fit, but the R^2^_PRE_ was 0.8852, reflecting limited generalization ability and slight overfitting. This model showed limited prediction performance and insufficient generalization on the current dataset. UVE–SPA selected 5 characteristic variables, the R^2^cv was 0.9606, and the R^2^_PRE_ reached 0.9705. The model demonstrated the best stability and prediction accuracy among all methods, suggesting its potential as a candidate approach for further validation in larger-scale studies (Figure 6C,D). ICO selected 345 variables. The R^2^cv was 0.8920, and the R^2^_PRE_ was 0.7845, indicating moderate fitting and prediction performance. The model showed moderate overall performance (Figure 6E,F). CARS selected 15 variables. The R^2^cv reached 0.9650, indicating excellent internal fit. However, the R^2^_PRE_ was only 0.1876, revealing severe overfitting and a near-total loss of generalization ability. This model showed poor prediction performance on the test set and was therefore not selected for further analysis (Figure 6G,H).

In summary, the UVE–SPA, MWPLS, and ICO algorithms all improved the performance of the pH near-infrared quantitative model to varying degrees, whereas the CARS algorithm showed severe overfitting under the current dataset, with the lowest R^2^_PRE_ (0.1876) among the four methods. Among them, the UVE–SPA model achieved the most favorable balance among fitting accuracy, predictive ability, and model complexity. Specifically, UVE–SPA identified five characteristic pH spectral bands: 5000–5100 cm^−1^, 6558 cm^−1^, 7370 cm^−1^, 7192 cm^−1^, and 7750–7950 cm^−1^. These five bands may correspond to the overtones and combination absorptions of O–H stretching and bending vibrations in carboxyl (–COOH) and hydroxyl (–OH) groups, as well as to the C–H stretching vibrations of the carbon chains in organic acid molecules.

**Figure 7:**
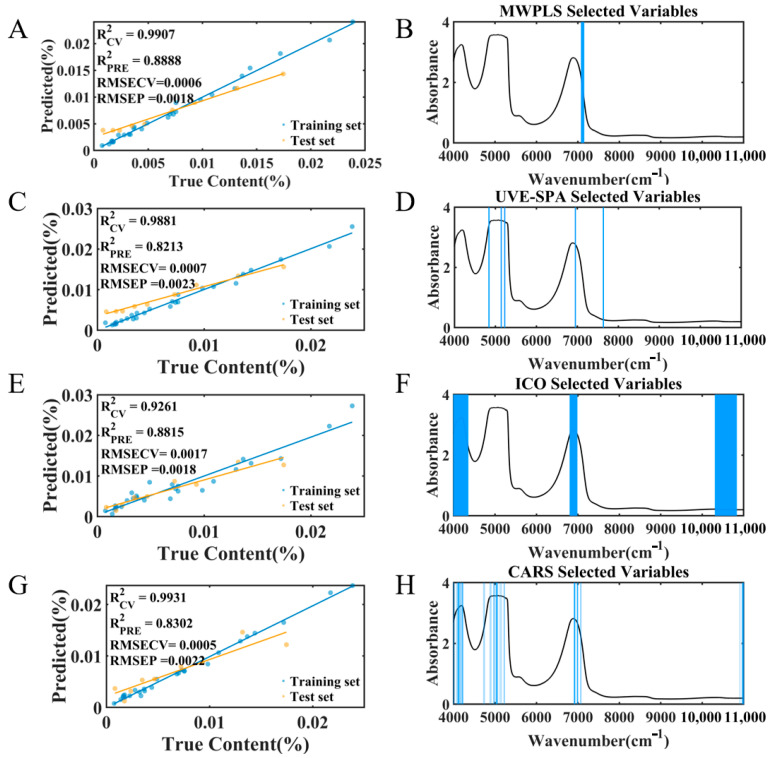
The regression fitting plots for the lycopene concentration model and the variable selection regions. (**A**,**B**) display the fitting plot and the corresponding variable selection region for MWPLS, (**C**,**D**) for UVE-SPA; (**E**,**F**) for ICO; and (**G**,**H**) for CARS.

As shown in Figure 7A,B, MWPLS identified 21 characteristic variables, giving the model an extremely parsimonious structure. The R^2^cv reached 0.9907, indicating excellent internal fit, and the R^2^_PRE_ was 0.8888, the highest among the four algorithms. At low complexity, this model achieved a favorable balance between fitting and prediction, despite a slight overfitting tendency. UVE–SPA selected only 5 key variables. The R^2^cv was 0.9881, and the R^2^_PRE_ was 0.8213. Its generalization performance was slightly inferior to that of MWPLS and ICO. Among the four algorithms, UVE–SPA used the fewest variables and produced the most parsimonious model, suggesting that UVE–SPA may be useful for developing simplified models with fewer variables (Figure 7C,D). ICO identified 339 characteristic variables. The R^2^cv was 0.9261, which was lower than those of MWPLS, UVE–SPA, and CARS. However, the R^2^_PRE_ reached 0.8815, indicating stable generalization performance and a low risk of overfitting (Figure 7E,F). CARS identified 53 characteristic variables. The R^2^cv reached 0.9931, the highest among the four models, indicating the best internal fit. However, the R^2^_PRE_ was only 0.8302 (Figure 7G,H), showing weaker generalization ability than MWPLS and ICO. CARS achieved the highest R^2^cv (0.9931) but the lowest R^2^_PRE_ (0.8302) among the four methods, indicating a pronounced overfitting tendency under the current dataset.

Overall, the MWPLS algorithm performed best for NIR detection of lycopene. Within the spectral range of 4000–11,000 cm^−1^, a spectral band at 7090–7150 cm^−1^ was selected by MWPLS and may be tentatively associated with lycopene. This band may be tentatively attributed to the first overtone of C–H stretching vibrations of methyl, methylene, and alkenyl C–H groups in lycopene molecules. However, in this complex aqueous beverage matrix, overlapping C–H absorption from other organic constituents (e.g., sugars, organic acids, and other carotenoids) may also contribute to the signal in this region. It may also contain vibrational contributions from conjugated C=C double bonds. Therefore, while this selected band is chemically plausible and provides a useful spectral variable basis for the NIR prediction model, its assignment should be regarded as tentative rather than definitive, given the spectral complexity of the beverage matrix.

## 4. Discussion

In this proof-of-concept study, we applied a combined preprocessing method of S-G smoothing and SNV transformation, and we compared four variable selection algorithms: ICO, UVE-SPA, CARS and MWPLS, to optimize models for pH, moisture, and lycopene content, as summarized in Table 4. For the original models, the R^2^_PRE_ of pH, moisture, and lycopene were 0.9276, 0.9951, and 0.8890, respectively. After SG-SNV preprocessing, these values changed to 0.9624, 0.9727, and 0.9576. The moisture model exhibited a slight decline but still maintained a high level. The combined preprocessing resulted in improved model performance for the pH and lycopene models under the current experimental conditions. Regarding the variable selection algorithms, the results indicate that the modeling performance of the four methods—MWPLS, UVE-SPA, ICO, and CARS—appears to vary with the target analyte. For pH, UVE-SPA appeared to be the most suitable method under the current experimental conditions. For moisture, CARS achieved the best internal fit, while ICO provided the highest prediction stability under the current dataset. For lycopene quantification, MWPLS yielded the highest prediction R^2^_PRE_ among the methods compared. Taken together, these results suggest that the proposed chemometric approach is a viable starting point warranting further investigation, rather than a ready-to-deploy method.

Furthermore, it should be noted that the spectral band assignments and SHAP-based interpretations presented here are inherently limited by the broad and overlapping nature of NIR absorption bands in complex aqueous matrices. Multiple beverage constituents—including sugars, organic acids, pigments, and water itself—share similar hydrogen-containing functional groups, and their NIR signals frequently overlap. Therefore, the spectral assignments in this study are best viewed as chemically plausible hypotheses that warrant confirmation through complementary techniques (e.g., spiking experiments, two-dimensional correlation spectroscopy, or targeted mid-IR analysis) rather than as definitive molecular attributions.

Compared with previous studies, our pH model achieved a prediction R^2^ of 0.9705 (UVE-SPA). As an informal reference, this value is higher than R^2^ values reported for several other beverage matrices—for example, pomegranate juice pH (R^2^ = 0.86) by Arendse et al., dragon fruit juice (R^2^ = 0.433) by Sinaga et al., and apple juice (R^2^ = 0.733) by Włodarska et al.—although direct comparison is limited by differences in sample matrices, reference methods, and spectrometer configurations [50,51,52]. In related work, a siPLS-GA approach has also been applied to pH determination in wine fermentation broth [53]. For moisture, our ICO model achieved an R^2^ of 0.9930, which is higher than the value (R^2^ = 0.88) reported by Otani et al. for roasted and ground coffees, although the two studies differ substantially in sample matrix, spectrometer type, and experimental design [54]. For lycopene, our MWPLS model achieved an R^2^ of 0.8888, which exceeds the random forest machine learning prediction (R^2^ = 0.79) reported by Fass et al. for pre-harvest tomato fruit quality, and is slightly below the PLSR result (R^2^ = 0.9175) reported by Deak et al. for processed tomato homogenate, although the datasets, modeling approaches, and validation strategies differ across these studies [55,56]. These cross-study comparisons are provided only as a general reference and should not be interpreted as rigorous benchmarking.

Several limitations of the current study should be acknowledged. First, the sample size was relatively limited, and the training and test sets had narrow coverage. These factors may impair the models’ generalisation ability and introduce a risk of overfitting. The applicability of the findings in complex real-world scenarios therefore requires further validation. Second, the samples were prepared using an artificial gradient design rather than collected from naturally occurring variation in commercial or field samples; as such, the present models may not fully capture the spectral variability encountered in real-world applications. Third, lycopene quantification in the present study was based on UV spectrophotometry. Despite its widespread adoption in comparable research, standard HPLC detection is still warranted as a confirmatory approach to guarantee the robustness of future large-scale data modeling and validation efforts. Finally, although external validation with an independent test set ensured a certain degree of analytical stability, all experimental samples were prepared from a limited number of production batches. Samples from different batches were included but batch diversity remained limited. Therefore, future studies should expand the sample sources and collect specimens from multiple batches and manufacturers for a second round of external validation, and should combine variable selection with model optimisation, to further evaluate model generalizability. Additionally, the severe overfitting observed for the CARS-pH model (R^2^_PRE_ = 0.1876) highlights the sensitivity of aggressive variable selection methods to small sample sizes and suggests that some algorithms evaluated here may require larger training sets to yield stable results. In summary, this study should be regarded as a proof-of-concept demonstration. While the approach shows potential for rapid, non-destructive multi-indicator detection, the small sample size, artificial gradient design, and limited batch diversity mean that the current models are not yet suitable for routine industrial application.

## 5. Conclusions

In this study, a preliminary NIR-based analytical approach for the simultaneous detection of pH, moisture content, and lycopene content was explored and evaluated under controlled laboratory conditions. Through combined spectral preprocessing and variable selection, the prediction performance was improved for pH and, to a lesser extent, for lycopene; for moisture, the original model already performed well and neither preprocessing nor variable selection yielded meaningful gains. In addition, the SHAP algorithm was introduced to partially address the “black box” limitation of traditional NIR models. The analysis identified positive and negative associations between individual spectral bands and the target components. This provided a chemically plausible, albeit tentative, explanation for model predictions from the perspective of vibrational spectroscopy, thereby offering a preliminary means to enhance the interpretability of spectral quantitative detection. However, given the overlapping and broad nature of NIR bands in aqueous beverage matrices, these spectral assignments should be interpreted with caution and regarded as suggestive rather than definitive. The results of this proof-of-concept study indicate that NIRS coupled with chemometric modeling shows potential for rapid multi-indicator detection of lycopene liquid beverages under laboratory conditions. However, extensive independent validation with larger and more diverse sample sets—ideally encompassing samples from different production batches and manufacturers—as well as production-line tests, are needed before the approach can be considered for routine implementation. Future work could explore adaptive preprocessing strategies, model transferability across instruments, validation with samples sourced from different production batches and manufacturers, and method validation with independent sample cohorts to further assess the generalizability of the proposed approach.

## Figures and Tables

**Table 1:** Statistical summary of pH, moisture content, and lycopene content of lycopene liquid beverages.

Components	Min	Max	Mean	SD
pH	3.95	4.26	4.12	0.07
Moisture Content (%)	84	99	94.78	4.6
Lycopene Content (%)	0.0753	2.3864	0.7176	0.6104

**Table 2:** Results of the original model.

Detection Indicator	PLSR							
	R^2^_CV_	RMSECV	RPD_C_	RERc	R^2^_PRE_	RMSEP	RPD_P_	RERp
pH	0.7592	0.0370	2.0767	9.4695	0.9276	0.0216	3.9424	12.9436
Moisture Content	0.9898	0.4614	10.1145	32.5069	0.9951	0.3095	15.1401	47.1732
Lycopene Content	0.8863	0.0021	3.0225	11.0226	0.8890	0.0018	3.1830	9.3340

**Table 3:** Optimal model results after SG-SNV preprocessing.

Detection Indicator	Parameters	PLSR							
		R^2^_CV_	RMSECV	RPD_C_	RERc	R^2^_PRE_	RMSEP	RPD_P_	RERp
pH	2, 7	0.9398	0.0185	4.1534	18.9388	0.9624	0.0156	5.4716	17.9619
Moisture Content	3, 7	0.9617	0.8959	5.2098	16.7437	0.9727	0.7301	6.4182	19.9976
Lycopene Content	1, 5	0.9860	0.0007	8.6277	31.4640	0.9576	0.0011	5.1482	15.0966

**Table 4:** Summary of model results combining optimal preprocessing with variable selection.

Components	Method	R^2^cv	RMSECV	RPDc	RERc	R^2^_PRE_	RMSEP	RPDp	RERp
Moisture content	MWPLS	0.9771	0.7792	5.9902	19.2517	0.8793	1.5348	3.0531	9.5128
	UVE-SPA	0.9660	0.8450	5.5236	17.7521	0.9397	1.0852	4.3178	13.4533
	ICO	0.9977	0.2182	21.3926	68.7532	0.9930	0.3705	12.6471	39.4056
	CARS	0.9992	0.1324	35.2607	113.3237	0.9946	0.3254	14.3994	44.8655
Lycopene Content	MWPLS	0.9907	0.0006	10.5393	38.4352	0.8888	0.0018	3.1804	9.3262
	UVE-SPA	0.9881	0.0007	9.3479	34.0903	0.8213	0.0023	2.5091	7.3576
	ICO	0.9261	0.0017	3.7497	13.6746	0.8815	0.0018	3.0814	9.0360
	CARS	0.9931	0.0005	12.2832	44.7950	0.8302	0.0022	2.5741	7.5483
pH	MWPLS	0.9486	0.0171	4.4948	20.4956	0.8852	0.0272	3.1304	10.2763
	UVE-SPA	0.9606	0.0150	5.1310	23.3963	0.9705	0.0138	6.1739	20.2674
	ICO	0.8920	0.0248	3.1004	14.1371	0.7845	0.0373	2.2846	7.4997
	CARS	0.9650	0.0141	5.4442	24.8245	0.1876	0.0725	1.1768	3.8632

## Data Availability

The original contributions presented in this study are included in the article. Further inquiries can be directed to the corresponding authors.
